# Cell Surface Profiling of Retinal Müller Glial Cells Reveals Association to Immune Pathways after LPS Stimulation

**DOI:** 10.3390/cells10030711

**Published:** 2021-03-23

**Authors:** Lea Lorenz, Sieglinde Hirmer, Adrian Schmalen, Stefanie M. Hauck, Cornelia A. Deeg

**Affiliations:** 1 Chair of Physiology, Department of Veterinary Sciences, LMU Munich, 82152 Martinsried, Germany; Lea.Lorenz@tiph.vetmed.uni-muenchen.de (L.L.); sieglinde.hirmer@tiph.vetmed.uni-muenchen.de (S.H.); 2 Research Unit Protein Science, Helmholtz Center Munich, German Research Center for Environmental Health (GmbH), 80939 Munich, Germany; adrian.schmalen@helmholtz-muenchen.de (A.S.); hauck@helmholtz-muenchen.de (S.M.H.)

**Keywords:** retinal Müller glial cells, MIO-M1, neuroinflammation, LPS, cell surface proteomics, LC-MS/MS, ocular inflammation, immune capacity of RMG, atypical APC

## Abstract

Retinal Müller glial cells (RMG) are involved in virtually every retinal disease; however, the role of these glial cells in neuroinflammation is still poorly understood. Since cell surface proteins play a decisive role in immune system signaling pathways, this study aimed at characterizing the changes of the cell surface proteome of RMG after incubation with prototype immune system stimulant lipopolysaccharide (LPS). While mass spectrometric analysis of the human Müller glia cell line MIO-M1 revealed 507 cell surface proteins in total, with 18 proteins significantly more abundant after stimulation (ratio ≥ 2), the surfaceome of primary RMG comprised 1425 proteins, among them 79 proteins with significantly higher abundance in the stimulated state. Pathway analysis revealed notable association with immune system pathways such as “antigen presentation”, “immunoregulatory interactions between a lymphoid and a non-lymphoid cell” and “cell migration”. We could demonstrate a higher abundance of proteins that are usually ascribed to antigen-presenting cells (APCs) and function to interact with T-cells, suggesting that activated RMG might act as atypical APCs in the course of ocular neuroinflammation. Our data provide a detailed description of the unstimulated and stimulated RMG surfaceome and offer fundamental insights regarding the capacity of RMG to actively participate in neuroinflammation in the retina.

## 1. Introduction

In recent years, retinal Müller glial cells (RMG) have gained increasing attention, as they are involved in virtually every retinal disease and represent valuable therapeutic targets (reviewed in [1,2,3,4]). RMG are the principal macroglial cells of the retina [5] and span its entire thickness from the inner to the outer limiting membrane [6]. Thus, they are in close contact to the vitreous, the subretinal space and retinal blood vessels, while they also enclose all retinal neurons with their cell processes [1,7]. Under physiological conditions, RMG are involved in maintaining retinal water-, pH- and ion- homeostasis [7,8], and they contribute to the blood–retinal barrier (BRB) with their endfeet [9]. Furthermore, they support retinal neurons by recycling neurotransmitters [5,10] and supplying them with nutrients [1,11,12]. In the case of retinal trauma or disease, they undergo a phenotypical transformation and become gliotic [13]. Gliosis is regarded as a cellular attempt to impede further tissue damage after an insult or to repair affected retinal tissue [14]. It has been demonstrated that, under pathological conditions, RMG actively participate in the immune response, acting as antigen-presenting cells (APCs) and releasing various pro-inflammatory cytokines [15,16,17,18,19]. However, little is known about their exact role in ocular immune response and the consequences of their activation. We previously showed that RMG were activated in the course of autoimmune uveitis in horses, resulting in IFN-γ expression and the downregulation of cell-specific proteins such as glutamine-synthetase (GS), aquaporins 5 and 11 and potassium channel Kir4.1, thus playing a detrimental role in disease pathogenesis [19,20,21,22]. In order to gain further insight into the capacity of RMG to transform into an activated phenotype upon a danger signal, we carried out an in-depth analysis of the RMG surfaceome after stimulation with prototype immune system stimulant lipopolysaccharide (LPS). In our study, we performed a glycocapture proteomics approach, which allowed us to selectively analyze the RMG surfaceome. Since cell surface proteins carry out important functions, such as receptor signaling, communication between cells and cell adhesion (reviewed in [23,24,25]), they play a crucial role in immune system signaling pathways. For our experiments, we used liquid chromatography coupled to tandem mass spectrometry (LC-MS/MS), which has proven to be a powerful tool for the in-depth analysis of cell surface proteins [26,27,28]. In order to compare the reactive capacity of primary RMG and a Müller glia cell line, we performed our mass spectrometric analysis on both primary equine RMG and the permanent human Müller glia cell line MIO-M1 [29]. Our results demonstrate that the RMG surfaceome changes considerably in response to immune stimulation and thus provide fundamental knowledge about the contribution of RMG to immune system signaling.

## 2. Materials and Methods

### 2.1. Sample Collection and Preparation

For this study, four control eyes from healthy horses were collected from a local abattoir. Collection and use of equine eyes from the abattoir were approved for purposes of scientific research by the appropriate board of the veterinary inspection office, Munich, Germany. No experimental animals were involved in this study. Eyes were processed immediately after enucleation. After removal of residual periocular tissue, eyeballs were rinsed in 70% ethanol followed by washing in cold PBS and storing in DMEM until further use. Preparation of primary RMG was conducted under a laminar flow hood with sterile instruments as previously described [30]. Briefly, eyeballs were cut open circumferentially parallel to the limbus corneae, and anterior parts of the eyes were removed. Retina was carefully detached from the vitreous and the attached pigment epithelium and mechanically disintegrated using microscissors. Resulting fragments were enzymatically digested at 37 °C with papain that had been activated by incubation with 1.1 µM EDTA, 0.067 µM mercaptoethanol and 5.5 µM cysteine-HCl prior to use. Enzymatical digestion was stopped after 30 min by adding Dulbecco’s modified Eagles Medium (DMEM, Pan Biotech, Aidenbach, Germany) with 10% fetal bovine serum (FBS, Biochrom, Darmstadt, Germany). After addition of Desoxyribonuclease I (Sigma-Aldrich Chemie GmbH, Taufkirchen, Germany), cells were first triturated and then collected by centrifugation (100× *g*, 10 min). Subsequently, cells were resuspended and seeded into 25 cm^2^ tissue flasks (Sarstedt, Nümbrecht, Germany) with DMEM containing 10% FBS and 1% penicillin/streptomycin (Pan Biotech). After allowing the cells to adhere for 24 h, thorough panning of the flasks followed by removal of the supernatant was performed in order to eliminate nonattached neuronal cells. The efficiency of this method to yield pure RMG cultures has been demonstrated previously [30,31]. Cells were cultured at 37 °C and 5% CO_2_ with repeated medium exchange until reaching confluence. Phase-contrast microscopy was used to determine the extent of confluence as well as to monitor the purity of growing cell population by verification of homologous morphology as previously described [29,30]. The human Müller cell line Moorfields/Institute of Ophthalmology- Müller 1 (MIO-M1) was obtained from G. A. Limb [29] and grown to approximately 80% confluency at 37 °C and 5% CO_2_ with repeated medium exchanges.

### 2.2. Stimulation of Cells and Biotinylation of Cell Surface Proteins

For the following experiments, seven T75 flasks with pooled adherent primary equine RMG or MIO-M1 cells were used. For both cell types, three of them were used for stimulation with LPS (Sigma-Aldrich), and cells of three flasks served as negative controls, whereas the cells of the remaining flask were not biotinylated and thus were used as a negative control for biotinylation. Prior to stimulation with LPS, cells were washed with phosphate buffered saline (PBS) and incubated at 37 °C in DMEM without FBS for starvation. After 2 h, supernatant was removed and replaced with fresh DMEM not containing FBS. LPS was added to three flasks at a final concentration of 10 µg/mL. Negative controls were incubated at the same conditions without addition of LPS. After 24 h at 37 °C, supernatant was removed, and cells were washed twice with PBS—the first time with prewarmed PBS (pH 7.4) and the second time with PBS with an adjusted pH of 6.7, containing 1 mM CaCl_2_ and 0.5 mM MgCl_2_. Cell surface membrane proteins were then labelled with 2 mL of biotinylation reagent per flask, containing 309.6 µL aminooxy-biotin (VWR, International GmbH, Darmstadt, Germany), 24 µL NaIO_4_ and 11.04 µL aniline (Sigma-Aldrich) in labeling buffer (PBS, pH 6.7) and incubated in the dark for 30 min at 4 °C. The biotinylation reaction was quenched by adding glycerol to a final concentration of 1 mM for an additional 5 min at 4 °C. Subsequently, medium was removed, and cells were washed with washing buffer (PBS with 1 mM CaCl_2_ and 0.5 mM MgCl_2_, pH 7.4) before scraping the cells carefully into 500 µL lysis buffer (1% Nonidet P-40, 150 mM NaCl, 10 mM Tris-HCl (pH 7.4), a 1× complete protease inhibitor mixture, EDTA-free (Roche, Grenzach-Wyhlen, Germany) in HPLC-grade water) and immediately frozen at −20 °C.

### 2.3. Preparation of Plasma Membrane Fraction and Protein Extraction from Affinity-Purified Plasma Membrane

To enrich biotinylated cell surface and cell membrane fractions, lysates were thawed on ice for 30 min and vortexed before centrifugation at 16,000× *g* at 4 °C for 10 min. The supernatant was diluted 1:5 with washing buffer, and the pellet was discarded. Streptavidin beads (IBA GmbH, Göttingen, Germany) were prewashed 3 times with lysis buffer diluted 1:5 with washing buffer and centrifuged between the washing steps at 1000× *g* for 1 min. Then, samples were added to the washed streptavidin beads in LoBind tubes (Eppendorf, Hamburg, Germany) and incubated at 4 °C for 2 h on a rotator for binding of biotinylated proteins to high-affinity streptavidin beads. Subsequently, extensive washing and incubation steps were performed, each in a volume of 500 µL to remove nonspecifically bound proteins. Centrifugation was carried out at 2500× *g* for 2 min as part of the washing process. First, beads were washed with lysis buffer diluted 1:5 with washing buffer, followed by a washing step with 0.5% SDS in washing buffer and incubation with 0.5% SDS and 100 mM DTT in washing buffer for 30 min at room temperature. Next, beads were washed with UC buffer (6 M urea, 100 mM Tris-Hcl, pH 8.5) and subsequently incubated with UC buffer containing 50 mM iodoacetamide for 30 min at room temperature for alkylation. This was followed by washing steps in UC buffer, 5 M NaCl, 100 mM Na_2_CO_3_ (pH 11.5) and 50 mM Tris-HCl (pH 8.5). For proteolysis, bead–protein complexes were incubated in 100 µL Tris-HCl (pH 8.5) containing 2.5 µg sequencing grade modified trypsin (Promega, Walldorf, Germany) at 37 °C overnight. Subsequently, the tryptic peptides were collected by centrifugation at 2000× *g* for 2 min before beads were resuspended in 50 µL 50 mM Tris-HCl (pH 8.5), followed by another centrifugation step. Supernatants resulting from both steps with the tryptic peptides therein were pooled together into a new LoBind tube (Eppendorf) and acidified with trifluoroacetic acid (TFA). To elute the glycopeptides, the beads were washed with 100 µL 1× G7 buffer and then incubated in 50 µL 1× G7 buffer containing 500 U glycerol-free PNGase F (New England Biolabs, Frankfurt a. Main, Germany) for 6 h at 37 °C, followed by centrifugation. Resulting supernatant was transferred into a new LoBind tube, and beads were resuspended in 50 µL 1× G7 buffer and centrifuged again. Resulting eluates from both steps were pooled in LoBind tubes (Eppendorf) and dried. Finally, dried glycopeptides were resolved in the acidified tryptic peptides and analyzed as combined samples by LC-MS/MS.

### 2.4. Mass Spectrometric Analysis

LC-MS/MS analysis of RMG surfaceomes was performed on an Ultimate3000 nano-HPLC system (Dionex, Sunnyvale, CA) online coupled to a LTQ OrbitrapXL mass spectrometer (Thermo Fisher Scientific, Bremen, Germany) by a nanospray ion source. Acidified eluted fractions were loaded automatically to a HPLC system equipped with a nanotrap column, eluted after 5 min and separated on the analytical column (75 µm inner diameter × 25 cm, Acclaim PepMap100 C18, 3 µm, 100 Å) by a 265 min gradient flow at a flow rate of 300 nL/min from 2 to 40 percent ACN. Peptides were analyzed by the LTQ OrbitrapXL, recording MS spectra at a resolution of 60,000 in profile mode. From the high-resolution MS prescan, the ten most intense peptide ions were selected for fragment analysis in the linear ion trap if they exceeded an intensity of at least 200 counts and if they were at least doubly charged. The normalized collision energy for CID was set to a value of 35, and the resulting fragments were detected with normal resolution in the linear ion trap in centroid mode. Dynamic exclusion was set to 60 s.

### 2.5. Data Analysis

Acquired MS spectral files were imported to Progenesis QI software (Nonlinear Dynamics, Waters, Newcastle upon Tyne, UK), and label-free quantitative analysis was performed as previously described [32,33]. All MS/MS spectra were exported from Progenesis QI software as Mascot generic files (mgf) and searched against Ensembl horse or human protein database, respectively (https://www.ensembl.org, accessed on 18 March 2021), for peptide identification with Mascot (https://www.matrixscience.com/, accessed on 18 March 2021). Search parameters used were 10 ppm peptide mass tolerance, one Da fragment mass tolerance and one missed cleavage allowed. Iodoacetamide derivatives of cysteines were set as stable modifications, and methionine oxidation as well as asparagine and glutamine deamidation were allowed as variable modifications. A Mascot integrated decoy database search calculated an average false discovery rate < 1% when searches were performed with a Mascot percolator cut-off score of 13 and with an appropriate significance threshold (*p*). Identifications were re-imported into Progenesis QI. Detailed information on all identified proteins is provided in Supplementary Tables S1 and S2.

### 2.6. Data Processing

Proteins were considered differentially abundant when LPS/unstimulated control ratio was at least 2. For statistical analysis, transformed normalized abundances were used for one-way analysis of variance (ANOVA) calculations. The volcano plots were created with OriginPro 2020 software (Additive, Friedrichsdorf, Germany). Pathway enrichment analyses were performed on human orthologs of gene names using open-source software ShinyGO (v0.61, http://bioinformatics.sdstate.edu/go/, accessed on 18 March 2021) with the following settings: search species human, *p*-value cutoff (FDR) 0.05, number of most significant terms to show 30. The *p*-value for enrichment analysis was calculated via hypergeometric distribution followed by FDR correction. Voronoi visualization of pathway analyses was conducted using open-source software Reactome (v75, https://reactome.org, accessed on 18 March 2021). In MIO-M1 cells, 2 identifiers could not be found in Reactome (CD82 and PODXL), whereas in primary RMG, 9 proteins could not be identified and thus were not included in pathway analyses, namely, CD82, CLMP, DMRTC2, HIRIP3, LRPAP1, MGARP, TMEM33, TSPAN6 and VASN.

## 3. Results

### 3.1. Mass Spectrometric Analysis Reveals 507 Cell Surface Proteins in Human Müller Glia Cell Line MIO-M1 and 1425 in Primary RMG

Using mass spectrometry, we identified 507 cell surface proteins in cells of the human retinal Müller glia cell line MIO-M1 (Supplementary Table S1) and 1425 proteins in the surfaceome of primary equine RMG (Supplementary Table S2).

### 3.2. Stimulation with LPS Results in Distinct Changes of RMG Surface Proteome

**Figure 1:**
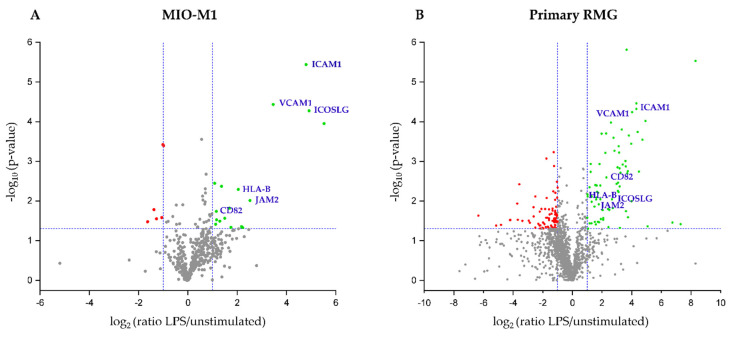
Volcano plot of all identified proteins in (**A**) MIO-M1 cells and (**B**) primary retinal Müller glial cells (RMG). (**A**) In MIO-M1 cells, 507 proteins were identified. Of those, 18 showed significantly (*p*-value ≤ 0.05) higher abundance (fold change ≥ 2, green dots) after stimulation with LPS, while six proteins showed significantly lower abundance following LPS stimulation (fold change < 0.5, red dots). (**B**) of all 1425 identified proteins in primary RMG, 79 identifications were significantly more abundant (green dots), whereas 82 proteins were significantly less abundant (red dots) after LPS stimulation. Dotted blue lines indicate cut-off values for p-value and ratio. Proteins that were more abundant after LPS in both primary RMG and MIO-M1 cells are labeled with their human orthologue gene name in blue.

To assess the capacity of RMG to respond to immune stimuli, MIO-M1 cells as well as primary RMG were stimulated with LPS for 24 h. Mass spectrometry revealed distinct changes of protein abundances after LPS stimulation (Table 1 and Figure 1). For further analyses, a two-fold change in abundance after stimulation with LPS was set as the cut-off value, and protein abundance change was regarded as significant at *p* ≤ 0.05. Following these criteria, we identified 18 proteins with significantly higher abundance after LPS stimulation in the MIO-M1 cell line (Table 1 and Figure 1A, green dots) and six proteins that showed lower abundance after co-incubation with LPS (Figure 1A, red dots). In contrast, the cell surfaceome of primary RMG comprised 79 identifications with higher abundance after LPS (Table 1 and Figure 1B, green dots), among them four proteins that belong to the MHC class Ⅰ family, while 82 proteins showed significantly lower abundance after in vitro stimulation (Figure 1B, red dots). While there was no overlap between primary RMG and MIO-M1 cells within the less abundant proteins, six candidates were significantly more abundant concomitantly in both cell types (Figure 1A,B, labeled with their gene names in blue font). These proteins comprised the leukocyte antigen Class I-B (HLA-B), inducible T-cell co-stimulatory ligand (ICOSLG), the tetraspanin CD82 and adhesion molecules intercellular adhesion molecule 1 (ICAM1), vascular cell adhesion molecule 1 (VCAM1) and junctional adhesion molecule 2 (JAM2), all of which have a function in immune system signaling.

### 3.3. Proteins with Higher Abundance in Stimulated RMG Associated with Immune System Pathways

**Figure 2:**
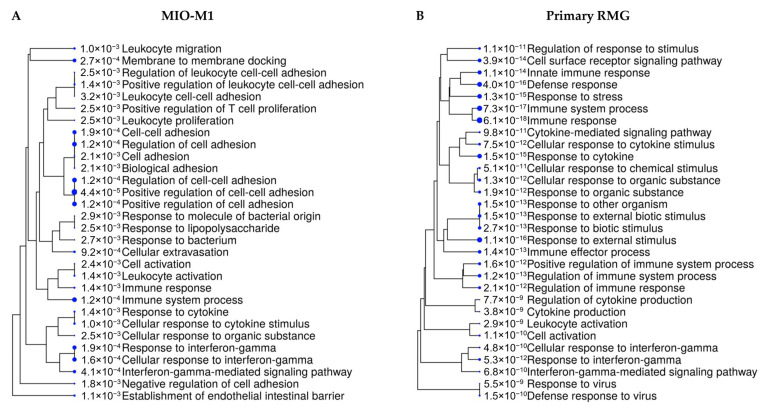
Shiny GO enrichment tree showing the 30 most significantly enriched functional categories from biological processes after LPS stimulation in (**A**) MIO-M1 cells and (**B**) primary RMG. Pathway enrichment analyses were performed with human orthologue gene names of proteins with significantly higher abundance in stimulated state. Size of the solid blue dots corresponds to the enrichment FDR with bigger dots indicating more significant *p*-values. Pathways with many shared genes are clustered together. Gene names of the proteins which clustered to respective pathways are given in Supplementary Table S3.

To take a closer look at the reactions of RMG to prototype immune system stimulant LPS, we selected proteins with significantly higher abundance after LPS stimulation in the MIO-M1 cell line and in primary RMG for pathway analyses. Enrichment analysis using open-source software ShinyGO revealed a strong association with immune system pathways (Figure 2). Proteins with higher abundance in stimulated MIO-M1 cells were allocated primarily to cell-adhesion processes (Figure 2A), whereas proteins of primary RMG clustered predominantly to the pathways “immune system process”, “cellular response to stimulus” as well as “cell surface receptor and cytokine signaling” (Figure 2B).

**Figure 3:**
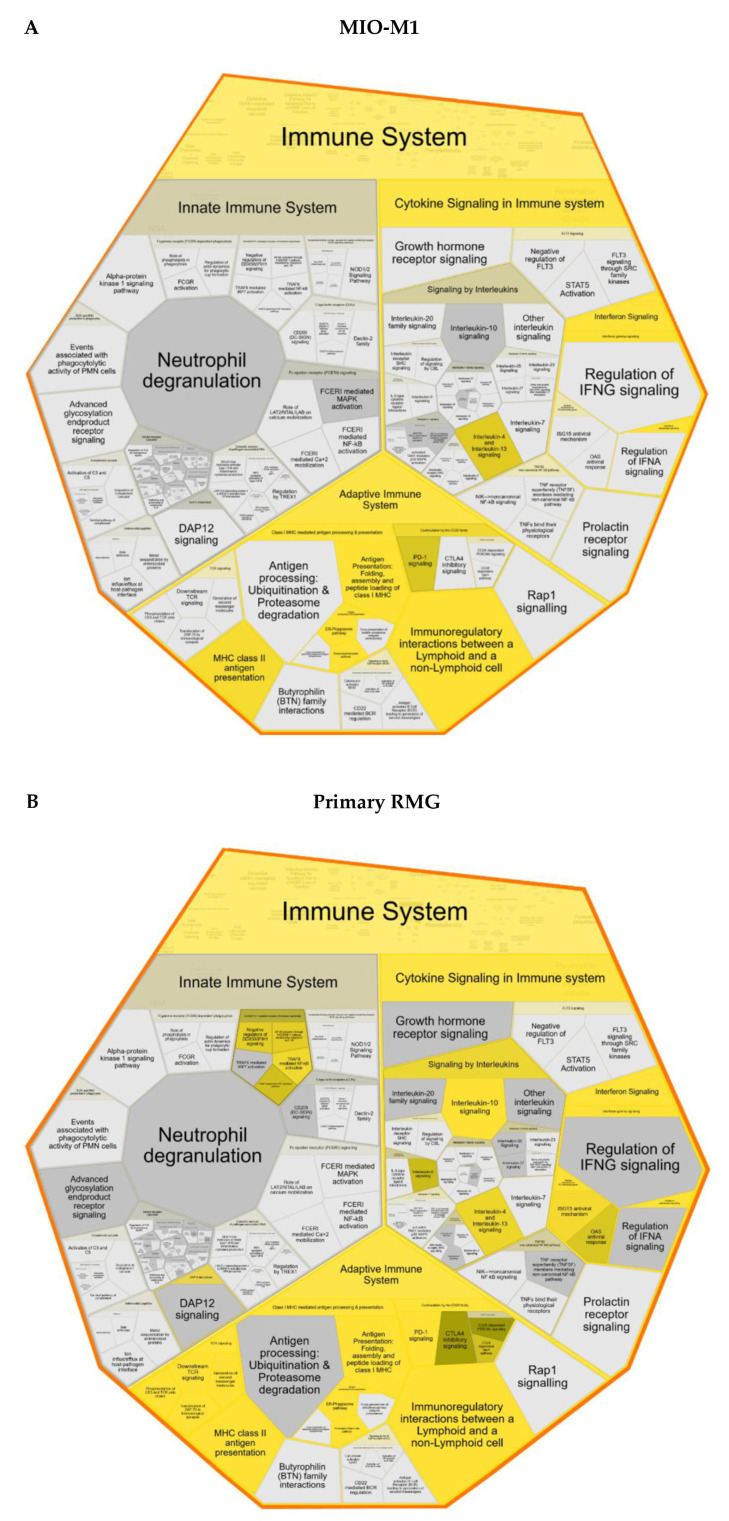
Voronoi diagram illustrating reactome analysis results comparing enriched pathways after LPS stimulation in (**A**) MIO-M1 cells and (**B**) primary retinal Müller glial cells (RMG). Enlarged polygon represents reactome superpathway immune system (for overview see Supplementary Figure S1 and Figure S2). Pathway enrichment analysis was calculated with human orthologue gene names of proteins with significantly higher abundance after LPS. Color intensity displays *p*-value of the statistical test for overrepresentation as illustrated by the color bar. Polygons colored in dark grey represent pathways without significant overrepresentation. Pathways without assigned proteins are displayed in light grey.

Furthermore, reactome pathway analysis confirmed associations with all three major subsets of the reactome super pathway immune system (“innate immune system”, “adaptive immune system” and “cytokine signaling in the immune system”) both for MIO-M1 cells and primary RMG (Figure 3). Overlap of enriched pathways between both cell types was especially pronounced among the adaptive immune system pathways. Proteins with higher abundance after stimulation in both cell types were allocated amongst others to the pathways “MHC class II antigen presentation”, “immunoregulatory interactions between a lymphoid and a non-lymphoid cell” and “co-stimulation by the CD28 family”, including “PD-1 signaling”, but also to “interleukin-4 and -13 and interferon signaling” (Figure 3A,B). Among the pathways which were enriched solely in proteins of primary cells, we detected “TCR signaling”, but also pathways of the innate immune system (“DDX58/IFIH1-mediated induction of interferon-alpha/beta”) and pathways belonging to the cytokine signaling system (“interleukin-6 and -10 signaling” as well as “ISG15 antiviral mechanism” and “OAS1 antiviral response”) (Figure 3B). There were no pathways exclusively overrepresented in the MIO-M1 cell line.

## 4. Discussion

The data we present here clearly demonstrate the capacity of RMG to respond to immune stimuli in a highly differentiated manner. We found that those RMG surface proteins which were more abundant after LPS stimulation clustered to all three major subsets of the reactome super pathway immune system with a focus on adaptive immunity and cytokine signaling, but also to cell adhesion processes. This is in line with a previous study, which showed that stimulation with LPS caused the nuclear translocation of NFκB in primary RMG of C57BL/6J mice as well as cytokine release, and that media from LPS-stimulated RMG promoted leukocyte adhesion and endothelial migration [34]. Here, we provide a proteomic in-depth analysis of RMG surfaceome dynamics to in vitro LPS stimulation. With this hypothesis-generating approach, we were able to provide an unambiguous and much more detailed analysis of RMG surface proteins compared to antibody-based methods. Since our aim was to analyze and describe the reaction of the RMG surfaceome to nonspecific immune stimulation, we used prototype immune system stimulant LPS for our experiments. Our finding that cell-surface proteins of LPS-stimulated RMG clearly cluster to immune system pathways indicates an important role of RMG in general immune system processes and lays the basis for further investigations. Based on our proteomic dataset, further studies need to be carried out using various cytokines to gain more detailed insight in the reactive capacity of RMG. Since LPS represents a classical danger signal for the immune system in various species [35,36,37], the reaction of Müller cells to this stimulatory signal might be of interest for further studies concerning gene therapies of ocular diseases. Genome editing causes unpredictable side effects due to the stimulation of an immune response by the delivery system for the genome editing vectors [38]. Thus, the capacity of Müller cells to actively participate in the immune response needs to be studied thoroughly in order to prevent unwanted side effects. In our analyses, we could demonstrate higher expression levels of OAS1 and ISG15 in primary RMG after LPS stimulation (Table 1) concomitant with the overrepresentation of the pathways “OAS1 antiviral response” and “ISG15 antiviral mechanism” (Figure 3B). This finding is of great interest, since ISG15 can function as an extracellular cytokine, interacting with various immune cells, such as natural killer cells, macrophages, dendritic cells and neutrophils, by modulating their function, but additionally, ISG15 is capable of inhibiting viral replication (reviewed in [39]). ISG15 expression is strongly induced by type I interferons, and its induction by LPS was also reported [40]. Thus, higher expression levels of ISG15 point to an activation of RMG after LPS stimulation.

To our knowledge, we were the first to perform mass spectrometric analysis on LPS-stimulated RMG surface proteins using both the spontaneously immortalized human RMG cell line MIO-M1 and primary cells. MIO-M1 cells have been shown to express known RMG markers such as cellular retinaldehyde binding protein (CRALBP) and GS. Furthermore, MIO-M1 cells depolarize upon glutamate treatment, indicating that the MIO-M1 cell line maintains the functional phenotype of RMG [29]. However, it is known that cell lines can change their phenotype over multiple passages and lose tissue-specific functions as they adapt to in vitro conditions [41]. In the case of MIO-M1, it has been observed that these cells express marker genes of neural progenitors as well as postmitotic neuronal cells and various opsins [42,43]. Thus, primary cells are often considered as more physiologically similar to in vivo cells [44]. Therefore, we also analyzed primary equine RMG. We previously demonstrated that cultured equine RMG express cell-specific proteins, such as GS and vimentin [30]. Thus, they represent a valuable model to study RMG functions in vitro. Our results revealed several proteins and pathways that were overrepresented after LPS stimulation in both MIO-M1 cells and primary RMG and, therefore, seem to represent a fundamental response pattern of RMG in retinal neuroinflammation. However, primary cells displayed a much more complex pathway and protein profile, which supports the importance of primary cells for studying the characteristics of RMG in vivo. For the preparation of primary RMG, we used equine retina, as control eyes from healthy horses are readily available, and retinal inflammation in the equine eye has proven to be a valuable model for glial involvement in neuroinflammation [19,20,21,45]. While interpreting our results, we must take into account that, in contrast to the vascularized human retina, the equine retina is almost completely avascular [46]. Except for a small area around the optic disc, the equine retina is devoid of both blood vessels and astrocytes [46,47]. Therefore, in contrast to human retinal tissue, RMG are the only macroglial cells in the equine retina [1]. In human retina, RMG contribute to the inner BRB, as they surround retinal blood vessels with their cell processes [7,48], whereas in horses, they are involved in the outer BRB, which separates the retina from the underlying pigment epithelium and choroidea [45,47]. This might account for some of the differences between MIO-M1 cells and equine RMG that we observed in this study. For instance, pathway enrichment analysis demonstrated cell-adhesion and migration processes as highly overrepresented, especially in MIO-M1 cells (Figure 3). This is an intriguing finding considering that human RMG cell processes closely surround retinal blood vessels and might, therefore, be implicated in the migration of cells into retinal tissue.

One main finding of this study was the expression of proteins, which are usually ascribed to APCs and are necessary for their interaction with T-cells, in RMG. MHC class I and II molecules were prominent among the proteins associated with antigen presentation and cell–cell interactions. “MHC class I antigen presentation” was overrepresented both in MIO-M1 cells and primary RMG after LPS stimulation (Figure 3), and MHC class I molecules were more abundant after LPS stimulation in both cell types (Table 1), confirming the LPS-induced pro-inflammatory activation of RMG. Furthermore, in primary RMG, β-2-microglobulin (B2M), which represents another subunit of the MHC-complex, and Tap binding protein (TAPBP), which has an important function in peptide loading on MHC-I molecules [49], were significantly more abundant after stimulation (Table 1). Since MHC-I molecules are essential for antigen presentation to CD8 T-cells, which play a pivotal role in neuroinflammatory diseases [50,51,52], this finding merits further investigation from our point of view. In line with this, we demonstrated a higher abundance of MHC class II molecules in primary RMG but not in the MIO-M1 cell line after stimulation (Table 1). MHC class II molecules are primarily expressed by professional APCs, which process and present antigen to CD4 T-cells. In combination with co-stimulatory signals, this leads to T-cell activation [53]. In ocular inflammation, MHC class II-associated antigen presentation and T-cell activation were primarily ascribed to invading macrophages and resident microglia, but not to RMG [54,55]. However, the expression of MHC class II molecules in human RMG has been reported in a case of subretinal fibrosis and uveitis syndrome concomitant with T-helper-cell infiltration [18]. Furthermore, in equine recurrent uveitis, the expression of MHC class II molecules in RMG has been described within retinal glial scars, whereas the surrounding parts of the retina revealed no MHC class II expression [56]. The data we present here confirm the ability of RMG to express MHC class II molecules upon immune stimulation.

Moreover, we detected the pathways “immunoregulatory interactions between a lymphoid and a non-lymphoid cell” and “co-stimulation by the CD28 family”, as overrepresented pathways that are associated with interaction with T-cells (Figure 3). Among the proteins allocated to these pathways was ICOSLG, which was more abundant in both MIO-M1 cells and primary RMG (Table 1 and Figure 1). To our knowledge, ICOSLG expression has not been described in RMG before. Its higher expression in response to LPS stimulation is an interesting finding, since ICOSLG is primarily expressed on APCs, such as human monocyte derived dendritic cells [57], and its binding to ICOS serves as a co-stimulatory signal for activated T-cells in humans [57]. In a murine model of autoimmune uveitis, the in vivo blocking of IGOSLG signaling reduced disease severity and led to a smaller number of inflammatory infiltrates in the retina, suggesting that ICOS-ICOSLG interaction plays a critical role in uveitis pathogenesis [58]. Considering this, we propose that the induction of ICOSLG expression in RMG upon immune stimulation might play a critical role in the course of neuroinflammation, as it could lead to co-stimulatory signals for T-cells invading the retina. This result is complemented by the finding of a higher abundance of the tetraspanin CD82 in LPS-stimulated RMG (Table 1 and Figure 1). As far as we know, there are no data on CD82 expression and function in RMG to date. This tetraspanin, which is expressed in both APCs and T-cells, plays a role in T-cell co-stimulation by inducing an enhanced interaction between APCs and T-cells, especially during the early phase of activation [59]. This is carried out through a stronger interaction between integrin β2 on T-cells and ICAM1 on APCs [60]. Thus, the elevated CD82 expression, which we detected in LPS-stimulated RMG, strengthens the hypothesis that RMG might act as atypical APCs that are able to interact with activated T-cells in the course of neuroinflammation in the retina. The implications of these interactions need to be addressed in future studies. Interestingly, we found that several other proteins which are involved in T-cell co-stimulation were also more abundant after LPS stimulation in primary RMG [61,62,63]. These include CD40 (Table 1), which has been described in RMG before [64,65], but also CD80 and CD86 (Table 1), whose ocular expression was associated with microglial cells [66] but not with RMG. Taken together, these results suggest the ability of RMG to interact with lymphocytes via different receptors and thus point to a pivotal role of these cells in T-cell mediated neuroinflammation. Therefore, further investigations concerning the role of respective proteins in inflammatory diseases of the eye need to be carried out.

Further proteins with higher abundance after LPS that belonged to the pathway “co-stimulation by the CD28 family” were programmed cell death 1 ligand 2 (PDCD1LG2) in MIO-M1 cells and CD274 (programmed cell death 1 ligand 1, PD-L1) in primary RMG (Table 1). Similar to CD80 and CD86, these proteins belong to the B7 family. Since they bind to the immunoinhibitory receptor PD1 on activated human T-cells, they inhibit lymphocyte proliferation and cytokine secretion [67]. Thus, they are potent players in the maintenance of tolerance and the prevention of autoimmunity [68]. PD-L1 is primarily expressed on APCs [68], but it has also been detected in murine RMG [69], and its expression levels have been shown to be higher in inflamed ocular tissue [70]. Moreover, ocular cells increase the abundance of PD-L1 and PD-L2 after stimulation by IFN-γ [70], which is in line with our finding of higher expression levels of PD-L1 and PD-L2 after LPS stimulation in RMG. The upregulation of PD-ligands might represent a regulatory mechanism that counteracts inflammatory pathways. Thus, our findings reveal that, in addition to their proinflammatory role, RMG might also exert a protective function in neuroinflammation by resolving ocular immune responses and maintaining retinal homoeostasis. Further investigations are required to elucidate the mechanisms of immunoregulation exerted by RMG.

Since little is known about the involvement of RMG in leukocyte migration to date, our finding that pathways such as “cell adhesion”, “cellular extravasation” and “leukocyte migration” were overrepresented in RMG after stimulation with LPS (Figure 2) is intriguing. Among the proteins allocated to these processes were ICAM1 and VCAM1 (Supplementary Table S3). Both proteins were more abundant in stimulated MIO-M1 cells as well as in stimulated primary RMG (Table 1 and Figure 1), which is in line with previous findings of increased ICAM1 expression after stimulation with LPS or IFN-γ in murine and rat Müller cells [71,72]. ICAM1 mediates the adhesion and migration of leukocytes through the endothelial barrier into inflamed tissue (reviewed in [73,74,75]), and it has been shown to regulate the migration of Th1- and Th17- T-cells through simulated human retinal endothelium [76]. High expression levels of these adhesion molecules have been reported in posterior uveitis in humans [77] and in murine EAU with VCAM1 expression in the perivascular extensions of RMG in uveitic murine eyes [78]. Moreover, the contribution of RMG to the regulation of leukocyte adhesion and migration has been proposed in a rat model of retinal diode laser photocoagulation, as ICAM1 was induced in RMG cell processes that surrounded the infiltrated CD4 T-cells and macrophages [79]. Thus, our result of significantly higher ICAM1 and VCAM1 expression levels in MIO-M1 cells and equine RMG after LPS stimulation point towards a regulatory role of RMG in leukocyte migration.

This is further supported by the finding of JAM2 (more often referred to as JAM-B, which will be used in the following) among the significantly more abundant proteins after LPS stimulation in both cell types (Table 1 and Figure 1). To our knowledge, we are the first to demonstrate that LPS-stimulated RMG express elevated levels of this adhesion molecule, which plays a role in leukocyte adhesion and migration [80]. At this point, it is noteworthy that the blockade of JAM-B in a murine model of CNS autoimmunity was accompanied by a partial protection against encephalomyelitis and a significant reduction in the number of infiltrating CD8 T-cells [81]. In this model, JAM-B was expressed in CNS endothelial cells and has been proposed as a ligand for the α4β1-integrin-mediated migration of CD8 T-cells into the CNS [81]. Taking this into account, we suggest that the higher expression levels of ICAM1, VCAM1 and JAM-B in activated RMG might render them capable of attracting and binding activated lymphocytes, thus contributing to lymphocyte homing into the inflamed retina. Since RMG are in close contact to retinal blood vessels in the human retina [7], this might represent a mechanism which enables lymphocytes to enter retinal tissue under inflammatory conditions.

## 5. Conclusions

In conclusion, our results provide a detailed description of the untreated and LPS-stimulated RMG surfaceome and strongly suggest an involvement of RMG in neuroinflammatory processes in the retina. The capacity to express proteins involved in antigen presentation and leukocyte migration might enable these glial cells to interact with invading lymphocytes, thus affecting the course of neuroinflammation. Since we used different cell types for our experiments, those candidates which were more abundant after LPS stimulation in both models might represent a common pathway in activated RMG. With our hypothesis-generating approach, we lay the basis for further investigations regarding the role of RMG in neuroinflammatory diseases.

## Figures and Tables

**Table 1:** Proteins from mass spectrometry dataset with statistically significant (*p* ≤ 0.05) abundance changes (ratio ≥ 2) after LPS stimulation (1) in MIO-M1 cells and (2) in primary retinal Müller glial cells (RMG).

Protein ID	Description	Gene Name	Peptides Used for Quantification	*p*-Value	Ratio LPS/Unstim
**(1) MIO-M1**					
ENSP00000006053	C-X3-C motif chemokine ligand 1	CX3CL1	4	0	46
ENSP00000339477	**Inducible T cell costimulator ligand**	**ICOSLG**	9	0	30.1
ENSP00000264832	**Intercellular adhesion molecule 1**	**ICAM1**	29	0	27.7
ENSP00000294728	**Vascular cell adhesion molecule 1**	**VCAM1**	47	0	11
ENSP00000318416	**Junctional adhesion molecule 2**	**JAM2**	1	0.01	5.7
ENSP00000166534	Prolyl 4-hydroxylase subunit alpha 2	P4HA2	2	0.047	4.7
ENSP00000412429	Transgelin 2	TAGLN2	2	0.045	4.5
ENSP00000399168	**Major histocompatibility complex, class I, B**	**HLA-B**	2	0.005	4.1
ENSP00000304592	Fatty acid synthase	FASN	1	0.047	3.4
ENSP00000336799	Tubulin alpha 1b	TUBA1B	2	0.015	3.2
ENSP00000341289	Tubulin beta 4B class IVb	TUBB4B	2	0.028	2.8
ENSP00000240095	Solute carrier family 39 member 14	SLC39A14	13	0.004	2.6
ENSP00000330054	Eukaryotic translation elongation factor 1 alpha 1	EEF1A1	3	0.033	2.4
ENSP00000307046	Syndecan 2	SDC2	3	0.03	2.2
ENSP00000227155	**CD82 molecule**	**CD82**	8	0.018	2.2
ENSP00000245185	Metallothionein 2A	MT2A	1	0.039	2.2
ENSP00000319782	Podocalyxin like	PODXL	10	0.004	2.1
ENSP00000380855	**Programmed cell death 1 ligand 2**	**PDCD1LG2**	2	0.021	2
**(2) Primary RMG**					
ENSECAP00000013197	**TAP binding protein**	**TAPBP**	1	0	infinity
ENSECAP00000010634	SLAM family member 7	SLAMF7	1	0.039	158.6
ENSECAP00000009810	5,-nucleotidase, cytosolic IIIA	NT5C3A	1	0.035	107.7
ENSECAP00000009600	Transmembrane protein 33	TMEM33	1	0.044	33.8
ENSECAP00000018879	Neuregulin 1	NRG1	2	0	30.5
ENSECAP00000014003	**CD274 molecule**	**CD274**	2	0	26.4
ENSECAP00000019916	Betacellulin	BTC	1	0.002	22.4
ENSECAP00000000924	**ISG15 ubiquitin like modifier**	**ISG15**	7	0	21.1
ENSECAP00000011996	**Intercellular adhesion molecule 1**	**ICAM1**	25	0	19.9
ENSECAP00000009944	**CD86 molecule**	**CD86**	7	0	19.9
ENSECAP00000014290	**Vascular cell adhesion molecule 1**	**VCAM1**	51	0	16.3
ENSECAP00000004905	*Doublesex- and mab-3-related transcription factor C2*	*DMRTC2*	1	0.01	15.9
ENSECAP00000017760	Neuromedin U receptor 2	NMUR2	2	0	15.7
ENSECAP00000011171	**2,-5,-oligoadenylate synthetase 1**	**OAS1**	2	0	14
ENSECAP00000000811	**Beta-2-microglobulin**	**B2M**	1	0.026	13.4
ENSECAP00000010646	MX dynamin like GTPase 2	MX1	8	0.002	13.4
ENSECAP00000021590	***MHC class I heavy chain***	***HLA-A***	3	0.002	13.1
ENSECAP00000018982	Guanylate binding protein 5	GBP5	10	0	12.6
ENSECAP00000009324	Serum amyloid A1	SAA1	3	0.018	12.2
ENSECAP00000020078	***MHC class I heavy chain***	***HLA-A***	11	0.001	12.2
ENSECAP00000006405	Interferon induced with helicase C domain 1	IFIH1	22	0.001	12
ENSECAP00000020119	Very low-density lipoprotein receptor	VLDLR	1	0.001	10.5
ENSECAP00000003048	Interferon-induced protein with tetratricopeptide repeats 1	IFIT1	6	0	10
ENSECAP00000009264	**MHC class I heavy chain**	***HLA-A***	7	0.002	9.5
ENSECAP00000008591	ATP binding cassette subfamily A member 1	ABCA1	1	0.048	9.2
ENSECAP00000015028	Carbonic anhydrase 12	CA12	3	0.004	9.1
ENSECAP00000016719	Pleckstrin	PLEK	3	0.002	9
ENSECAP00000019669	DExD/H-box helicase 58	DDX58	18	0.001	8.9
ENSECAP00000002356	***MHC class I heavy chain***	***HLA-A***	1	0	8.7
ENSECAP00000006671	Semaphorin 4A	SEMA4A	2	0.006	8.7
ENSECAP00000022282	Intercellular adhesion molecule 3	ICAM3	3	0.003	8.5
ENSECAP00000017347	Versican	VCAN	4	0.006	8.4
ENSECAP00000014907	**CD40 molecule**	**CD40**	8	0.001	8.2
ENSECAP00000000620	Galectin 3 binding protein	LGALS3BP	2	0.004	7.9
ENSECAP00000007529	DnaJ heat shock protein family (Hsp40) member C3	DNAJC3	1	0.001	7.2
ENSECAP00000012605	HIRA interacting protein 3	HIRIP3	1	0	6.8
ENSECAP00000010541	**MHC class II DR alpha chain**	**HLA-DRA**	2	0.015	6.6
ENSECAP00000006847	**Inducible T cell costimulator ligand**	**ICOSLG**	4	0.009	6.4
ENSECAP00000008012	Interferon induced protein with tetratricopeptide repeats 3	IFIT3	3	0	6.1
ENSECAP00000017943	Syndecan 4	SDC4	4	0.017	5.6
ENSECAP00000017562	CXADR like membrane protein	CLMP	3	0.046	5.4
ENSECAP00000003412	Major prion protein	PRNP	2	0.016	5.3
ENSECAP00000017893	**CD82 molecule**	**CD82**	7	0.003	4.9
ENSECAP00000015940	C-X-C motif chemokine ligand 16	CXCL16	1	0	4.8
ENSECAP00000019909	**MHC class II DR-beta chain**	**HLA-DRB1**	1	0.001	4.7
ENSECAP00000018161	**MHC class I heavy chain**	***HLA-C***	2	0.03	4.2
ENSECAP00000006146	Praja ring finger ubiquitin ligase 2	PJA2	2	0.027	4.2
ENSECAP00000010650	DnaJ heat shock protein family (Hsp40) member C8	DNAJC8	1	0.017	4.1
ENSECAP00000001275	Carcinoembryonic antigen-related cell adhesion molecule 1	CEACAM21	9	0	3.9
ENSECAP00000015833	Lumican	LUM	1	0.029	3.8
ENSECAP00000018257	CD38 molecule	CD38	2	0.009	3.7
ENSECAP00000017093	Versican	VCAN	5	0.004	3.7
ENSECAP00000002944	Interferon induced protein with tetratricopeptide repeats 5	IFIT5	7	0.001	3.6
ENSECAP00000016198	Folate receptor beta	FOLR2	1	0.034	3.5
ENSECAP00000012733	**Junctional adhesion molecule 2**	**JAM2**	2	0.012	3.4
ENSECAP00000011113	Guanylate-binding protein 6	GBP6	9	0.006	3.3
ENSECAP00000007853	Signal transducer and activator of transcription 1	STAT1	14	0.034	3.3
ENSECAP00000014392	Endothelin converting enzyme 1	ECE1	17	0.006	3.2
ENSECAP00000002864	Vasorin	VASN	16	0.004	3.1
ENSECAP00000009001	Integrin subunit alpha 4	ITGA4	9	0.008	3.1
ENSECAP00000015309	Colony stimulating factor 1	CSF1	5	0.009	3
ENSECAP00000002058	Sphingosine-1-phosphate receptor 3	S1PR3	2	0.039	3
ENSECAP00000001010	*Hydroxycarboxylic acid receptor 2*	*HCAR2*	2	0.004	2.9
ENSECAP00000019459	Mitochondria localized glutamic acid rich protein	MGARP	1	0.034	2.9
ENSECAP00000001529	**CD80 molecule**	**CD80**	1	0.014	2.8
ENSECAP00000017208	Solute carrier family 1 member 3	SLC1A3	12	0.008	2.5
ENSECAP00000012239	Solute carrier family 20 member 1	SLC20A1	2	0.01	2.5
ENSECAP00000019326	Tissue factor pathway inhibitor	TFPI	2	0.037	2.4
ENSECAP00000019780	**MHC class I heavy chain**	***HLA-B***	7	0.007	2.4
ENSECAP00000005675	Plasminogen activator, urokinase	PLAU	5	0.001	2.4
ENSECAP00000000171	Semaphorin 7A	SEMA7A	1	0.002	2.4
ENSECAP00000011356	CD53 molecule	CD53	2	0.037	2.2
ENSECAP00000012737	LDL receptor related protein associated protein 1	LRPAP1	3	0.005	2.2
ENSECAP00000008846	*Epidermal growth factor receptor*	EGFR	22	0.05	2.1
ENSECAP00000002079	CD68 molecule	CD68	2	0.008	2
ENSECAP00000006770	Tetraspanin 6	TSPAN6	2	0.007	2
ENSECAP00000016635	CD47 molecule	CD47	5	0.026	2
ENSECAP00000019515	Mannose-6-phosphate receptor, cation dependent	M6PR	2	0.015	2
ENSECAP00000004902	Slit guidance ligand 3	SLIT3	4	0.007	2

Column 1 (protein ID) contains the accession number of the identified protein and column 2 (description) the respective protein name, both as listed in the Ensembl protein database (http://www.ensembl.org, accessed on 18 March 2021). Column 3 (gene name) contains the name of the human orthologue gene. Italicized names display proteins originally listed as uncharacterized proteins in the Ensembl database output or differently named as in later manual search. Searching by accession number in the Universal Protein Resource database (https://www.uniprot.org, accessed on 18 March 2021) or in the ncbi Basic Local Alignment Search Tool (https://blast.ncbi.nlm.nih.gov/Blast.cgi, accessed on 18 March 2021) provided the description for these proteins. Column 4 (peptides used for quantification) displays the number of unique peptides used for quantification. Column 5 shows the p-value as calculated by ANOVA. Column 6 (ratio) contains the fold change of the protein abundance after LPS stimulation compared to unstimulated controls. Proteins that are referred to in the text are illustrated in bold.

## Data Availability

Data are contained within the article or supplementary materials.

## Supplementary Materials

The following are available online at https://www.mdpi.com/2073-4409/10/3/711/s1, Figure S1: Reactome pathway analysis of significantly more abundant proteins in MIO-M1 cells after LPS stimulationl Figure S2: Reactome pathway analysis of significantly more abundant proteins in primary RMG after LPS stimulation; Table S1: All identified proteins of the MIO-M1 cell surfaceome; Table S2: All identified proteins of the primary RMG surfaceome; Table S3: Shiny GO enrichment analysis showing the most significantly enriched functional categories from biological processes after LPS stimulation in (1) MIO-M1 cells and (2) primary RMG including human orthologue gene names of proteins clustering to respective pathways.

## References

[1] Bringmann A., Pannicke T., Grosche J., Francke M., Wiedemann P., Skatchkov S.N., Osborne N.N., Reichenbach A. (2006). Müller cells in the healthy and diseased retina. Prog. Retin Eye Res..

[2] Ghaseminejad F., Kaplan L., Pfaller A.M., Hauck S.M., Grosche A. (2020). The role of Müller cell glucocorticoid signaling in diabetic retinopathy. Graefe’s Arch. Clin. Exp. Ophthalmol..

[3] Li X., Liu J., Hoh J., Liu J. (2019). Müller cells in pathological retinal angiogenesis. Transl. Res..

[4] Devoldere J., Peynshaert K., De Smedt S.C., Remaut K. (2019). Müller cells as a target for retinal therapy. Drug Discov. Today.

[5] Reichenbach A., Bringmann A. (2020). Glia of the human retina. Glia.

[6] Müller H. (1851). Zur histologie der netzhaut. Z. Wiss. Zool..

[7] Newman E., Reichenbach A. (1996). The Müller cell: A functional element of the retina. Trends Neurosci..

[8] Bringmann A., Reichenbach A., Wiedemann P. (2004). Pathomechanisms of cystoid macular edema. Ophthalmic Res..

[9] Tout S., Chan-Ling T., Holländer H., Stone J. (1993). The role of Müller cells in the formation of the blood-retinal barrier. Neuroscience.

[10] Matsui K., Hosoi N., Tachibana M. (1999). Active role of glutamate uptake in the synaptic transmission from retinal nonspiking neurons. J. Neurosci..

[11] Poitry-Yamate C.L., Poitry S., Tsacopoulos M. (1995). Lactate released by Müller glial cells is metabolized by photoreceptors from mammalian retina. J. Neurosci..

[12] Tsacopoulos M., Magistretti P.J. (1996). Metabolic coupling between glia and neurons. J. Neurosci..

[13] Bringmann A., Iandiev I., Pannicke T., Wurm A., Hollborn M., Wiedemann P., Osborne N.N., Reichenbach A. (2009). Cellular signaling and factors involved in Müller cell gliosis: Neuroprotective and detrimental effects. Prog. Retin Eye Res..

[14] Liberto C.M., Albrecht P.J., Herx L.M., Yong V.W., Levison S.W. (2004). Pro-regenerative properties of cytokine-activated astrocytes. J. Neurochem..

[15] Roberge F.G., Caspi R.R., Nussenblatt R.B. (1988). Glial retinal Müller cells produce IL-1 activity and have a dual effect on autoimmune T helper lymphocytes. Antigen presentation manifested after removal of suppressive activity. J. Immunol..

[16] Eastlake K., Banerjee P.J., Angbohang A., Charteris D.G., Khaw P.T., Limb G.A. (2016). Müller glia as an important source of cytokines and inflammatory factors present in the gliotic retina during proliferative vitreoretinopathy. Glia.

[17] Natoli R., Fernando N., Madigan M., Chu-Tan J.A., Valter K., Provis J., Rutar M. (2017). Microglia-derived IL-1β promotes chemokine expression by Müller cells and RPE in focal retinal degeneration. Mol. Neurodegener..

[18] Kim M.K., Chan C.C., Belfort R., Farah M., Burnier M.P., Nussenblatt R.B., Kuwabara T., Palestine A.G. (1987). Histopathologic and immunohistopathologic features of subretinal fibrosis and uveitis syndrome. Am. J. Ophthalmol..

[19] Hauck S.M., Schoeffmann S., Amann B., Stangassinger M., Gerhards H., Ueffing M., Deeg C.A. (2007). Retinal Mueller glial cells trigger the hallmark inflammatory process in autoimmune uveitis. J. Proteome Res..

[20] Eberhardt C., Amann B., Feuchtinger A., Hauck S.M., Deeg C.A. (2011). Differential expression of inwardly rectifying K+ channels and aquaporins 4 and 5 in autoimmune uveitis indicates misbalance in Müller glial cell-dependent ion and water homeostasis. Glia.

[21] Deeg C.A., Amann B., Lutz K., Hirmer S., Lutterberg K., Kremmer E., Hauck S.M. (2016). Aquaporin 11, a regulator of water efflux at retinal Muller glial cell surface decreases concomitant with immune-mediated gliosis. J. Neuroinflam..

[22] Lorenz L., Amann B., Hirmer S., Degroote R.L., Hauck S.M., Deeg C.A. (2021). NEU1 is more abundant in uveitic retina with concomitant desialylation of retinal cells. Glycobiology.

[23] Bachmann M., Kukkurainen S., Hytönen V.P., Wehrle-Haller B. (2019). Cell Adhesion by Integrins. Physiol. Rev..

[24] Gaud G., Lesourne R., Love P.E. (2018). Regulatory mechanisms in T cell receptor signalling. Nat. Rev. Immunol..

[25] Sweeney M.D., Zhao Z., Montagne A., Nelson A.R., Zlokovic B.V. (2019). Blood-Brain Barrier: From Physiology to Disease and Back. Physiol. Rev..

[26] Wu C.C., Yates J.R. (2003). The application of mass spectrometry to membrane proteomics. Nat. Biotechnol..

[27] Jeremiasse B., Matta C., Fellows C.R., Boocock D.J., Smith J.R., Liddell S., Lafeber F., van Spil W.E., Mobasheri A. (2020). Alterations in the chondrocyte surfaceome in response to pro-inflammatory cytokines. BMC Mol. Cell Biol..

[28] Uhl P.B., Szober C.M., Amann B., Alge-Priglinger C., Ueffing M., Hauck S.M., Deeg C.A. (2014). In situ cell surface proteomics reveals differentially expressed membrane proteins in retinal pigment epithelial cells during autoimmune uveitis. J. Proteom..

[29] Limb G.A., Salt T.E., Munro P.M., Moss S.E., Khaw P.T. (2002). In vitro characterization of a spontaneously immortalized human Müller cell line (MIO-M1). Invest. Ophthalmol. Vis. Sci..

[30] Eberhardt C., Amann B., Stangassinger M., Hauck S.M., Deeg C.A. (2012). Isolation, characterization and establishment of an equine retinal glial cell line: A prerequisite to investigate the physiological function of Muller cells in the retina. J. Anim. Physiol. Anim. Nutr. (Berl.).

[31] Hauck S.M., Suppmann S., Ueffing M. (2003). Proteomic profiling of primary retinal Müller glia cells reveals a shift in expression patterns upon adaptation to in vitro conditions. Glia.

[32] Hauck S.M., Hofmaier F., Dietter J., Swadzba M.E., Blindert M., Amann B., Behler J., Kremmer E., Ueffing M., Deeg C.A. (2012). Label-free LC-MSMS analysis of vitreous from autoimmune uveitis reveals a significant decrease in secreted Wnt signalling inhibitors DKK3 and SFRP2. J. Proteom..

[33] Hauck S.M., Dietter J., Kramer R.L., Hofmaier F., Zipplies J.K., Amann B., Feuchtinger A., Deeg C.A., Ueffing M. (2010). Deciphering Membrane-Associated Molecular Processes in Target Tissue of Autoimmune Uveitis by Label-Free Quantitative Mass Spectrometry. Mol. Cell Proteom..

[34] Shanmugam A., Wang J., Markand S., Perry R.L., Tawfik A., Zorrilla E., Ganapathy V., Smith S.B. (2015). Sigma receptor 1 activation attenuates release of inflammatory cytokines MIP1γ, MIP2, MIP3α, and IL12 (p40/p70) by retinal Müller glial cells. J. Neurochem..

[35] Iwami K.-I., Matsuguchi T., Masuda A., Kikuchi T., Musikacharoen T., Yoshikai Y. (2000). Cutting Edge: Naturally Occurring Soluble Form of Mouse Toll-Like Receptor 4 Inhibits Lipopolysaccharide Signaling. J. Immunol..

[36] Beutler B., Rietschel E.T. (2003). Innate immune sensing and its roots: The story of endotoxin. Nat. Rev. Immunol..

[37] Pacholewska A., Marti E., Leeb T., Jagannathan V., Gerber V. (2017). LPS-induced modules of co-expressed genes in equine peripheral blood mononuclear cells. BMC Genom..

[38] Chew W.L., Tabebordbar M., Cheng J.K.W., Mali P., Wu E.Y., Ng A.H.M., Zhu K., Wagers A.J., Church G.M. (2016). A multifunctional AAV–CRISPR–Cas9 and its host response. Nat. Methods.

[39] Perng Y.-C., Lenschow D.J. (2018). ISG15 in antiviral immunity and beyond. Nat. Rev. Microbiol..

[40] Malakhova O., Malakhov M., Hetherington C., Zhang D.E. (2002). Lipopolysaccharide activates the expression of ISG15-specific protease UBP43 via interferon regulatory factor 3. J. Biol. Chem..

[41] Pan C., Kumar C., Bohl S., Klingmueller U., Mann M. (2009). Comparative proteomic phenotyping of cell lines and primary cells to assess preservation of cell type-specific functions. Mol. Cell Proteom..

[42] Hollborn M., Ulbricht E., Rillich K., Dukic-Stefanovic S., Wurm A., Wagner L., Reichenbach A., Wiedemann P., Limb G.A., Bringmann A. (2011). The human Müller cell line MIO-M1 expresses opsins. Mol. Vis..

[43] Lawrence J.M., Singhal S., Bhatia B., Keegan D.J., Reh T.A., Luthert P.J., Khaw P.T., Limb G.A. (2007). MIO-M1 cells and similar muller glial cell lines derived from adult human retina exhibit neural stem cell characteristics. Stem Cells.

[44] Pereiro X., Ruzafa N., Acera A., Urcola A., Vecino E. (2020). Optimization of a Method to Isolate and Culture Adult Porcine, Rats and Mice Müller Glia in Order to Study Retinal Diseases. Front. Cell Neurosci.

[45] Deeg C.A., Eberhardt C., Hofmaier F., Amann B., Hauck S.M. (2011). Osteopontin and fibronectin levels are decreased in vitreous of autoimmune uveitis and retinal expression of both proteins indicates ECM re-modeling. PLoS ONE.

[46] Schnitzer J. (1988). Astrocytes in the guinea pig, horse, and monkey retina: Their occurrence coincides with the presence of blood vessels. Glia.

[47] Ehrenhofer M.C.A., Deeg C.A., Reese S., Liebich H.-G., Stangassinger M., Kaspers B. (2002). Normal structure and age-related changes of the equine retina. Vet. Ophthalmol..

[48] Reichenbach A., Wurm A., Pannicke T., Iandiev I., Wiedemann P., Bringmann A. (2007). Müller cells as players in retinal degeneration and edema. Graefe’s Arch. Clin. Exp. Ophthalmol..

[49] Blees A., Januliene D., Hofmann T., Koller N., Schmidt C., Trowitzsch S., Moeller A., Tampé R. (2017). Structure of the human MHC-I peptide-loading complex. Nature.

[50] Gross C.C., Meyer C., Bhatia U., Yshii L., Kleffner I., Bauer J., Tröscher A.R., Schulte-Mecklenbeck A., Herich S., Schneider-Hohendorf T. (2019). CD8+ T cell-mediated endotheliopathy is a targetable mechanism of neuro-inflammation in Susac syndrome. Nat. Commun..

[51] Unger M.S., Li E., Scharnagl L., Poupardin R., Altendorfer B., Mrowetz H., Hutter-Paier B., Weiger T.M., Heneka M.T., Attems J. (2020). CD8(+) T-cells infiltrate Alzheimer’s disease brains and regulate neuronal- and synapse-related gene expression in APP-PS1 transgenic mice. Brain Behav. Immun..

[52] Zhao Y., Qiu W., Liu J., Yuan X., Mao W., Yin J., Peng B., Liu W., Han S., He X. (2020). Blockade of Kv1.3 potassium channel inhibits CD8(+) T cell-mediated neuroinflammation via PD-1/Blimp-1 signaling. FASEB J..

[53] Guerder S., Flavell R.A. (1995). T-cell activation. Two for T. Curr. Biol..

[54] Lipski D.A., Dewispelaere R., Foucart V., Caspers L.E., Defrance M., Bruyns C., Willermain F. (2017). MHC class II expression and potential antigen-presenting cells in the retina during experimental autoimmune uveitis. J. Neuroinflam..

[55] Zhang J., Wu G.S., Ishimoto S., Pararajasegaram G., Rao N.A. (1997). Expression of major histocompatibility complex molecules in rodent retina. Immunohistochemical study. Invest. Ophthalmol. Vis. Sci..

[56] Romeike A., Brügmann M., Drommer W. (1998). Immunohistochemical studies in equine recurrent uveitis (ERU). Vet. Pathol..

[57] Aicher A., Hayden-Ledbetter M., Brady W.A., Pezzutto A., Richter G., Magaletti D., Buckwalter S., Ledbetter J.A., Clark E.A. (2000). Characterization of human inducible costimulator ligand expression and function. J. Immunol..

[58] Usui Y., Akiba H., Takeuchi M., Kezuka T., Takeuchi A., Hattori T., Okunuki Y., Yamazaki T., Yagita H., Usui M. (2006). The role of the ICOS/B7RP-1 T cell costimulatory pathway in murine experimental autoimmune uveoretinitis. Eur. J. Immunol..

[59] Shibagaki N., Hanada K., Yamaguchi S., Yamashita H., Shimada S., Hamada H. (1998). Functional analysis of CD82 in the early phase of T cell activation: Roles in cell adhesion and signal transduction. Eur. J. Immunol..

[60] Shibagaki N., Hanada K., Yamashita H., Shimada S., Hamada H. (1999). Overexpression of CD82 on human T cells enhances LFA-1/ICAM-1-mediated cell-cell adhesion: Functional association between CD82 and LFA-1 in T cell activation. Eur. J. Immunol..

[61] Chen L., Flies D.B. (2013). Molecular mechanisms of T cell co-stimulation and co-inhibition. Nat. Rev. Immunol..

[62] Karnell J.L., Rieder S.A., Ettinger R., Kolbeck R. (2019). Targeting the CD40-CD40L pathway in autoimmune diseases: Humoral immunity and beyond. Adv. Drug Deliv. Rev..

[63] Kelsall B.L., Stüber E., Neurath M., Strober W. (1996). Interleukin-12 Production by Dendritic Cells. Ann. N. Y. Acad. Sci..

[64] Portillo J.A., Okenka G., Kern T.S., Subauste C.S. (2009). Identification of primary retinal cells and ex vivo detection of proinflammatory molecules using flow cytometry. Mol. Vis..

[65] Portillo J.A., Greene J.A., Okenka G., Miao Y., Sheibani N., Kern T.S., Subauste C.S. (2014). CD40 promotes the development of early diabetic retinopathy in mice. Diabetologia.

[66] Louveau A., Nerrière-Daguin V., Vanhove B., Naveilhan P., Neunlist M., Nicot A., Boudin H. (2015). Targeting the CD80/CD86 costimulatory pathway with CTLA4-Ig directs microglia toward a repair phenotype and promotes axonal outgrowth. Glia.

[67] Freeman G.J., Long A.J., Iwai Y., Bourque K., Chernova T., Nishimura H., Fitz L.J., Malenkovich N., Okazaki T., Byrne M.C. (2000). Engagement of the PD-1 immunoinhibitory receptor by a novel B7 family member leads to negative regulation of lymphocyte activation. J. Exp. Med..

[68] Keir M.E., Butte M.J., Freeman G.J., Sharpe A.H. (2008). PD-1 and Its Ligands in Tolerance and Immunity. Annu. Rev. Immunol..

[69] Sham C.W., Chan A.M., Kwong J.M.K., Caprioli J., Nusinowitz S., Chen B., Lee J.G., Gandhi N.M., Francisco L.M., Sharpe A.H. (2012). Neuronal Programmed Cell Death-1 Ligand Expression Regulates Retinal Ganglion Cell Number in Neonatal and Adult Mice. J. Neuroophthalmol..

[70] Yang W., Li H., Chen P.W., Alizadeh H., He Y., Hogan R.N., Niederkorn J.Y. (2009). PD-L1 Expression on Human Ocular Cells and Its Possible Role in Regulating Immune-Mediated Ocular Inflammation. Invest. Ophthalmol. Vis. Sci..

[71] Drescher K.M., Whittum-Hudson J.A. (1996). Modulation of immune-associated surface markers and cytokine production by murine retinal glial cells. J. Neuroimmunol..

[72] Gu R., Ding X., Tang W., Lei B., Jiang C., Xu G. (2018). A Synthesized Glucocorticoid- Induced Leucine Zipper Peptide Inhibits Retinal Müller Cell Gliosis. Front. Pharm..

[73] Marchetti L., Engelhardt B. (2020). Immune cell trafficking across the blood-brain barrier in the absence and presence of neuroinflammation. VASC Biol..

[74] Ley K., Laudanna C., Cybulsky M.I., Nourshargh S. (2007). Getting to the site of inflammation: The leukocyte adhesion cascade updated. Nat. Rev. Immunol..

[75] Bui T.M., Wiesolek H.L., Sumagin R. (2020). ICAM-1: A master regulator of cellular responses in inflammation, injury resolution, and tumorigenesis. J. Leukoc. Biol..

[76] Bharadwaj A.S., Schewitz-Bowers L.P., Wei L., Lee R.W.J., Smith J.R. (2013). Intercellular Adhesion Molecule 1 Mediates Migration of Th1 and Th17 Cells Across Human Retinal Vascular Endothelium. Invest. Ophthalmol. Vis. Sci..

[77] Whitcup S.M., Chan C.-C., Li Q., Nussenblatt R.B. (1992). Expression of Cell Adhesion Molecules in Posterior Uveitis. Arch. Ophthalmol..

[78] Dewispelaere R., Lipski D., Foucart V., Bruyns C., Frère A., Caspers L., Willermain F. (2015). ICAM-1 and VCAM-1 are differentially expressed on blood-retinal barrier cells during experimental autoimmune uveitis. Exp. Eye Res..

[79] Richardson P.R., Boulton M.E., Duvall-Young J., McLeod D. (1996). Immunocytochemical study of retinal diode laser photocoagulation in the rat. Br. J. Ophthalmol..

[80] Weber C., Fraemohs L., Dejana E. (2007). The role of junctional adhesion molecules in vascular inflammation. Nat. Rev. Immunol..

[81] Martin-Blondel G., Pignolet B., Tietz S., Yshii L., Gebauer C., Perinat T., Van Weddingen I., Blatti C., Engelhardt B., Liblau R. (2015). Migration of encephalitogenic CD8 T cells into the central nervous system is dependent on the α4β1-integrin. Eur. J. Immunol..

