# Supplementary material for: Cell Surface Profiling of Retinal Müller Glial Cells Reveals Association to Immune Pathways after LPS Stimulation
Source: Cells. 2021 Mar 23;10(3):711. doi: 10.3390/cells10030711 (PMC8004686; doi:10.3390/cells10030711)
Supplement: Supplementary file 1 [file cells-10-00711-s001.zip › Supplementary/Supplementary Table S3.pdf]

**Supplementary Table 3.** Shiny GO enrichment analysis showing the most significantly enriched functional categories from biological processes after LPS stimulation in (1) MIO-M1 cells and (2) primary RMG including human orthologue gene names of proteins clustering to respective pathways.

| Enrichment FDR | Genes in list | Total genes | Functional Category | Genes |
|---|---|---|---|---|
| **1) MIO-M1** | | | | |
| 0.0001 | 6 | 471 | Regulation of cell-cell adhesion | PDCD1LG2 CX3CL1 PODXL VCAM1 ICAM1 ICOSLG |
| 0.0001 | 7 | 765 | Regulation of cell adhesion | PDCD1LG2 CX3CL1 PODXL VCAM1 ICAM1 JAM2 ICOSLG |
| 0.0001 | 6 | 465 | Positive regulation of cell adhesion | PDCD1LG2 CX3CL1 PODXL VCAM1 ICAM1 ICOSLG |
| 0.0001 | 12 | 3539 | Immune system process | CX3CL1 ICOSLG PDCD1LG2 VCAM1 HLA-B ICAM1 FASN TUBB4B MT2A JAM2 EEF1A1 SDC2 |
| 0.0002 | 5 | 289 | Cellular response to interferon-gamma | CX3CL1 ICAM1 MT2A VCAM1 HLA-B |
| 0.0002 | 7 | 922 | Cell-cell adhesion | PDCD1LG2 CX3CL1 PODXL VCAM1 ICAM1 JAM2 ICOSLG |
| 0.0002 | 5 | 312 | Response to interferon-gamma | CX3CL1 ICAM1 MT2A VCAM1 HLA-B |
| 0.0003 | 2 | 5 | Membrane to membrane docking | ICAM1 VCAM1 |
| 0.0004 | 4 | 178 | Interferon-gamma-mediated signaling pathway | ICAM1 MT2A VCAM1 HLA-B |
| 0.0009 | 3 | 73 | Cellular extravasation | VCAM1 CX3CL1 ICAM1 |
| 0.0010 | 5 | 491 | Leukocyte migration | CX3CL1 VCAM1 ICAM1 JAM2 SDC2 |
| 0.0010 | 7 | 1278 | Cellular response to cytokine stimulus | CX3CL1 ICAM1 TUBA1B VCAM1 FASN MT2A HLA-B |
| 0.0011 | 2 | 12 | Establishment of endothelial intestinal barrier | ICAM1 FASN |
| 0.0014 | 4 | 272 | Positive regulation of leukocyte cell-cell adhesion | PDCD1LG2 VCAM1 ICAM1 ICOSLG |
| 0.0014 | 7 | 1372 | Response to cytokine | CX3CL1 ICAM1 TUBA1B VCAM1 FASN MT2A HLA-B |
| 0.0014 | 9 | 2602 | Immune response | CX3CL1 ICOSLG PDCD1LG2 HLA-B ICAM1 TUBB4B MT2A EEF1A1 VCAM1 |
| 0.0014 | 7 | 1416 | Leukocyte activation | PDCD1LG2 VCAM1 CX3CL1 ICOSLG ICAM1 EEF1A1 TUBB4B |
| 0.0018 | 4 | 304 | Negative regulation of cell adhesion | PDCD1LG2 JAM2 PODXL CX3CL1 |
| 0.0021 | 7 | 1541 | Cell adhesion | ICAM1 VCAM1 PDCD1LG2 CX3CL1 PODXL JAM2 ICOSLG |
| 0.0021 | 7 | 1548 | Biological adhesion | ICAM1 VCAM1 PDCD1LG2 CX3CL1 PODXL JAM2 ICOSLG |
| 0.0024 | 7 | 1591 | Cell activation | PDCD1LG2 VCAM1 CX3CL1 ICOSLG ICAM1 EEF1A1 TUBB4B |
| 0.0025 | 4 | 363 | Regulation of leukocyte cell-cell adhesion | PDCD1LG2 VCAM1 ICAM1 ICOSLG |
| 0.0025 | 4 | 366 | Response to lipopolysaccharide | PDCD1LG2 CX3CL1 ICAM1 VCAM1 |
| 0.0025 | 3 | 136 | Positive regulation of T cell proliferation | PDCD1LG2 VCAM1 ICOSLG |

| Enrichment FDR | Genes in list | Total genes | Functional Category | Genes |
|---|---|---|---|---|
| 0.0025 | 4 | 360 | Leukocyte proliferation | PDCD1LG2 VCAM1 CX3CL1 ICOSLG |
| 0.0025 | 9 | 2938 | Cellular response to organic substance | CX3CL1 PDCD1LG2 ICAM1 EEF1A1 TUBA1B VCAM1 FASN MT2A HLA-B |
| 0.0027 | 5 | 718 | Response to bacterium | PDCD1LG2 CX3CL1 ICAM1 MT2A VCAM1 |
| 0.0029 | 4 | 385 | Response to molecule of bacterial origin | PDCD1LG2 CX3CL1 ICAM1 VCAM1 |
| 0.0032 | 4 | 399 | Leukocyte cell-cell adhesion | PDCD1LG2 VCAM1 ICAM1 ICOSLG |
| **2) Primary RMG** | | | | |
| 6.07E-04 | 42 | 2602 | Immune response | CD86 CD274 CD80 SEMA7A MX1 ICOSLG IFIH1 STAT1 GBP1 CXCL16 B2M IFIT1 ISG15 HLA-B HLA-A SLAMF7 OAS1 ICAM1 CD40 DDX58 IFIT3 IFIT5 PRNP CSF1 HLA-DRB1 SEMA4A HLA-DRA HLA-C TSPAN6 CD38 PJA2 CD68 GBP6 CD47 ICAM3 DNAJC3 TAPBP ITGA4 PLAU CD53 VCAM1 SAA1 |
| 7.27E-04 | 46 | 3539 | Immune system process | CD38 OAS1 CD86 IFIT3 CD274 CD80 SEMA7A IFIT5 MX1 ICOSLG CXCL16 CSF1 IFIT1 ITGA4 VCAM1 DDX58 IFIH1 STAT1 GBP1 B2M SAA1 HCAR2 ISG15 HLA-DRB1 CD47 HLA-DRA HLA-B HLA-A SLAMF7 ICAM1 CD40 DNAJC3 TAPBP PRNP SEMA4A HLA-C PLEK TSPAN6 SDC4 PJA2 CD68 GBP6 ICAM3 PLAU CD53 JAM2 |
| 1.09E-02 | 40 | 2561 | Response to external stimulus | OAS1 CD86 IFIT3 CD274 CD80 PLAU SEMA7A IFIT5 MX1 CXCL16 SAA1 IFIT1 SEMA4A CD47 TFPI DDX58 IFIH1 STAT1 SLIT3 CSF1 SLC1A3 ICAM1 CD40 DNAJC3 PLEK GBP1 NMUR2 EGFR NRG1 VCAM1 ABCA1 FOLR2 B2M ISG15 ITGA4 TSPAN6 CD68 GBP6 HLA-DRB1 VLDLR |
| 3.99E-02 | 36 | 2062 | Defense response | OAS1 IFIT3 SEMA7A IFIT5 MX1 IFIT1 CD47 DDX58 IFIH1 STAT1 GBP1 CXCL16 ISG15 HLA-A SLAMF7 ICAM1 CD40 DNAJC3 EGFR VCAM1 SAA1 CSF1 S1PR3 TSPAN6 PJA2 CD68 GBP6 HLA-DRB1 ICOSLG FOLR2 ICAM3 LGALS3BP B2M HLA-DRA HLA-C HLA-B |
| 1.31E-01 | 49 | 4507 | Response to stress | PLEK OAS1 IFIT3 PLAU SEMA7A IFIT5 MX1 PRNP IFIT1 CD47 TFPI DDX58 TMEM33 IFIH1 STAT1 GBP1 EGFR CXCL16 CSF1 ISG15 HLA-A CD38 SLAMF7 SLC1A3 ICAM1 CD40 DNAJC3 SDC4 NMUR2 VCAM1 VASN SAA1 S1PR3 ABCA1 TSPAN6 PJA2 CD68 MGARP GBP6 HLA-DRB1 ICOSLG FOLR2 ICAM3 LGALS3BP NRG1 B2M HLA-DRA HLA-C HLA-B |
| 1.50E-01 | 30 | 1372 | Response to cytokine | STAT1 GBP1 CD274 CXCL16 CSF1 IFIT1 ISG15 TFPI CD38 ICAM1 CD40 ITGA4 IFIT3 VCAM1 DDX58 IFIH1 SLIT3 CD47 GBP6 OAS1 CD86 CD80 MX1 B2M SAA1 HLA-DRB1 HLA-DRA HLA-C HLA-B HLA-A |

| Enrichment FDR | Genes in list | Total genes | Functional Category | Genes |
|---|---|---|---|---|
| 1.08E+00 | 28 | 1242 | Innate immune response | MX1 IFIH1 STAT1 GBP1 CXCL16 IFIT1 ISG15 HLA-A SLAMF7 OAS1 ICAM1 CD40 DDX58 IFIT3 IFIT5 CSF1 TSPAN6 PJA2 GBP6 CD47 ICAM3 VCAM1 B2M SAA1 HLA-DRB1 HLA-DRA HLA-C HLA-B |
| 3.90E+00 | 41 | 3287 | Cell surface receptor signaling pathway | TSPAN6 CD82 CD86 CD274 CD80 SEMA7A CD53 EGFR NRG1 ICOSLG VASN PRNP BTC SEMA4A ICAM1 STAT1 PLEK CSF1 CD40 ITGA4 VLDLR ABCA1 S1PR3 CD38 GBP1 SLIT3 CD47 ICAM3 OAS1 IFIT3 MX1 VCAM1 B2M SAA1 IFIT1 ISG15 HLA-DRB1 HLA-DRA HLA-C HLA-B HLA-A |
| 1.15E+01 | 32 | 1909 | Regulation of immune system process | CD38 CD86 CD274 CD80 SEMA7A ICOSLG CSF1 IFIH1 VCAM1 B2M HCAR2 CD47 HLA-B HLA-A CD40 DDX58 ITGA4 PRNP IFIT1 ISG15 STAT1 TSPAN6 ICAM1 GBP1 SDC4 PJA2 CD68 HLA-DRB1 SLAMF7 ICAM3 HLA-DRA HLA-C |
| 1.39E+01 | 28 | 1392 | Immune effector process | OAS1 IFIT3 SEMA7A IFIT5 MX1 IFIT1 DDX58 IFIH1 STAT1 B2M HLA-B HLA-A ICAM1 CD40 DNAJC3 GBP1 ISG15 SEMA4A CD47 TSPAN6 HLA-DRB1 SLAMF7 CD86 CD80 PLAU CD68 CD53 HLA-C |
| 1.49E+01 | 25 | 1054 | Response to external biotic stimulus | OAS1 CD86 IFIT3 CD274 CD80 IFIT5 MX1 IFIT1 DDX58 IFIH1 STAT1 TFPI ICAM1 CD40 DNAJC3 GBP1 VCAM1 ABCA1 B2M ISG15 CD47 TSPAN6 CD68 GBP6 HLA-DRB1 |
| 1.49E+01 | 25 | 1052 | Response to other organism | OAS1 CD86 IFIT3 CD274 CD80 IFIT5 MX1 IFIT1 DDX58 IFIH1 STAT1 TFPI ICAM1 CD40 DNAJC3 GBP1 VCAM1 ABCA1 B2M ISG15 CD47 TSPAN6 CD68 GBP6 HLA-DRB1 |
| 2.67E+01 | 25 | 1085 | Response to biotic stimulus | OAS1 CD86 IFIT3 CD274 CD80 IFIT5 MX1 IFIT1 DDX58 IFIH1 STAT1 TFPI ICAM1 CD40 DNAJC3 GBP1 VCAM1 ABCA1 B2M ISG15 CD47 TSPAN6 CD68 GBP6 HLA-DRB1 |
| 1.31E+02 | 37 | 2938 | Cellular response to organic substance | CD86 CD274 CD80 VASN PRNP ICAM1 TMEM33 STAT1 GBP1 EGFR CSF1 IFIT1 TFPI CD40 ITGA4 IFIT3 VCAM1 ABCA1 FOLR2 SLIT3 DDX58 IFIH1 CD47 CD68 MGARP GBP6 HLA-DRB1 OAS1 DNAJC3 MX1 B2M SAA1 ISG15 HLA-DRA HLA-C HLA-B HLA-A |
| 1.58E+02 | 26 | 1301 | Positive regulation of immune system process | CD38 CD86 CD274 CD80 SEMA7A ICOSLG CSF1 IFIH1 VCAM1 B2M HCAR2 CD47 HLA-B CD40 DDX58 ITGA4 PRNP ISG15 TSPAN6 ICAM1 STAT1 GBP1 PJA2 ICAM3 HLA-DRB1 HLA-DRA |
| 1.94E+02 | 40 | 3547 | Response to organic substance | CD86 CD274 CD80 VASN PRNP ICAM1 TMEM33 STAT1 GBP1 EGFR CXCL16 CSF1 IFIT1 ISG15 TFPI CD38 CD40 DNAJC3 DDX58 ITGA4 IFIT3 LUM VCAM1 ABCA1 FOLR2 |

| Enrichment FDR | Genes in list | Total genes | Functional Category | Genes |
|---|---|---|---|---|
| | | | | B2M SLIT3 IFIH1 CD47 CD68 MGARP GBP6 HLA-DRB1 OAS1 MX1 SAA1 HLA-DRA HLA-C HLA-B HLA-A |
| 2.14E+01 | 26 | 1325 | Regulation of immune response | SEMA7A ICOSLG IFIH1 B2M HLA-B HLA-A CD40 DDX58 PRNP TSPAN6 CD38 GBP1 CD274 PJA2 CD47 CD86 CD80 SLAMF7 ICAM3 ICAM1 ITGA4 STAT1 VCAM1 HLA-DRB1 HLA-DRA HLA-C |
| 5.30E+01 | 15 | 312 | Response to interferon-gamma | STAT1 GBP1 CXCL16 ICAM1 CD40 GBP6 CD47 OAS1 VCAM1 B2M HLA-DRB1 HLA-DRA HLA-C HLA-B HLA-A |
| 7.51E+02 | 25 | 1278 | Cellular response to cytokine stimulus | STAT1 GBP1 CSF1 IFIT1 TFPI ICAM1 CD40 ITGA4 IFIT3 VCAM1 SLIT3 CD47 GBP6 OAS1 CD86 CD80 MX1 B2M SAA1 ISG15 HLA-DRB1 HLA-DRA HLA-C HLA-B HLA-A |
| 1.07E+03 | 45 | 4820 | Regulation of response to stimulus | TSPAN6 SLC20A1 PLAU SEMA7A ICOSLG VASN PRNP SEMA4A CD47 PJA2 TFPI ICAM1 TMEM33 IFIH1 PLEK EGFR B2M SAA1 CSF1 HLA-B HLA-A CD40 DNAJC3 DDX58 NRG1 CXCL16 LRPAP1 BTC IFIT1 STAT1 CD38 GBP1 CD274 IFIT5 ABCA1 SLIT3 HLA-DRB1 CD86 CD80 SLAMF7 ICAM3 ITGA4 VCAM1 HLA-DRA HLA-C |
| 5.05E+03 | 38 | 3536 | Cellular response to chemical stimulus | CD86 CD274 CD80 CXCL16 VASN PRNP SAA1 ICAM1 TMEM33 STAT1 GBP1 EGFR CSF1 IFIT1 TFPI CD40 ITGA4 IFIT3 VCAM1 ABCA1 FOLR2 B2M SLIT3 DDX58 IFIH1 CD47 CD68 MGARP GBP6 HLA-DRB1 OAS1 DNAJC3 MX1 ISG15 HLA-DRA HLA-C HLA-B HLA-A |
| 9.78E+03 | 21 | 950 | Cytokine-mediated signaling pathway | STAT1 CSF1 SLIT3 OAS1 ICAM1 CD40 CD86 GBP1 IFIT3 CD80 MX1 VCAM1 B2M SAA1 IFIT1 ISG15 HLA-DRB1 HLA-DRA HLA-C HLA-B HLA-A |
| 1.08E+04 | 26 | 1591 | Cell activation | PLEK CD38 CD86 CD274 CD80 ITGA4 VCAM1 CD47 CD40 EGFR ICOSLG B2M PRNP SEMA4A ICAM1 SDC4 PJA2 HLA-DRB1 SLAMF7 SAA1 CSF1 DNAJC3 PLAU CD68 CD53 HLA-C |
| 1.51E+03 | 13 | 263 | Defense response to virus | OAS1 IFIT3 IFIT5 MX1 IFIT1 DDX58 IFIH1 STAT1 CD40 DNAJC3 GBP1 ISG15 TSPAN6 |
| 4.76E+04 | 13 | 289 | Cellular response to interferon-gamma | STAT1 GBP1 ICAM1 GBP6 CD47 OAS1 VCAM1 B2M HLA-DRB1 HLA-DRA HLA-C HLA-B HLA-A |
| 6.84E+04 | 11 | 178 | Interferon-gamma-mediated signaling pathway | STAT1 OAS1 ICAM1 GBP1 VCAM1 B2M HLA-DRB1 HLA-DRA HLA-C HLA-B HLA-A |
| 2.90E+05 | 23 | 1416 | Leukocyte activation | CD38 CD86 CD274 CD80 ITGA4 VCAM1 CD47 CD40 ICOSLG B2M PRNP SEMA4A ICAM1 SDC4 PJA2 HLA-DRB1 SLAMF7 CSF1 DNAJC3 PLAU CD68 CD53 HLA-C |

| Enrichment FDR | Genes in list | Total genes | Functional Category | Genes |
|---|---|---|---|---|
| 3.83E+05 | 19 | 925 | Cytokine production | SEMA7A DDX58 IFIH1 STAT1 CD274 B2M SAA1 CD40 LUM PRNP S1PR3 TSPAN6 GBP1 ABCA1 ISG15 CD47 HLA-DRB1 CD86 CD80 |
| 5.49E+05 | 13 | 356 | Response to virus | OAS1 IFIT3 IFIT5 MX1 IFIT1 DDX58 IFIH1 STAT1 CD40 DNAJC3 GBP1 ISG15 TSPAN6 |
| 7.73E+05 | 18 | 852 | Regulation of cytokine production | SEMA7A DDX58 IFIH1 STAT1 CD274 B2M SAA1 CD40 LUM PRNP S1PR3 TSPAN6 GBP1 ISG15 CD47 HLA-DRB1 CD86 CD80 |
