# Supplementary figures and images for: Cell Surface Profiling of Retinal Müller Glial Cells Reveals Association to Immune Pathways after LPS Stimulation
Source: Cells. 2021 Mar 23;10(3):711. doi: 10.3390/cells10030711 (PMC8004686; doi:10.3390/cells10030711)

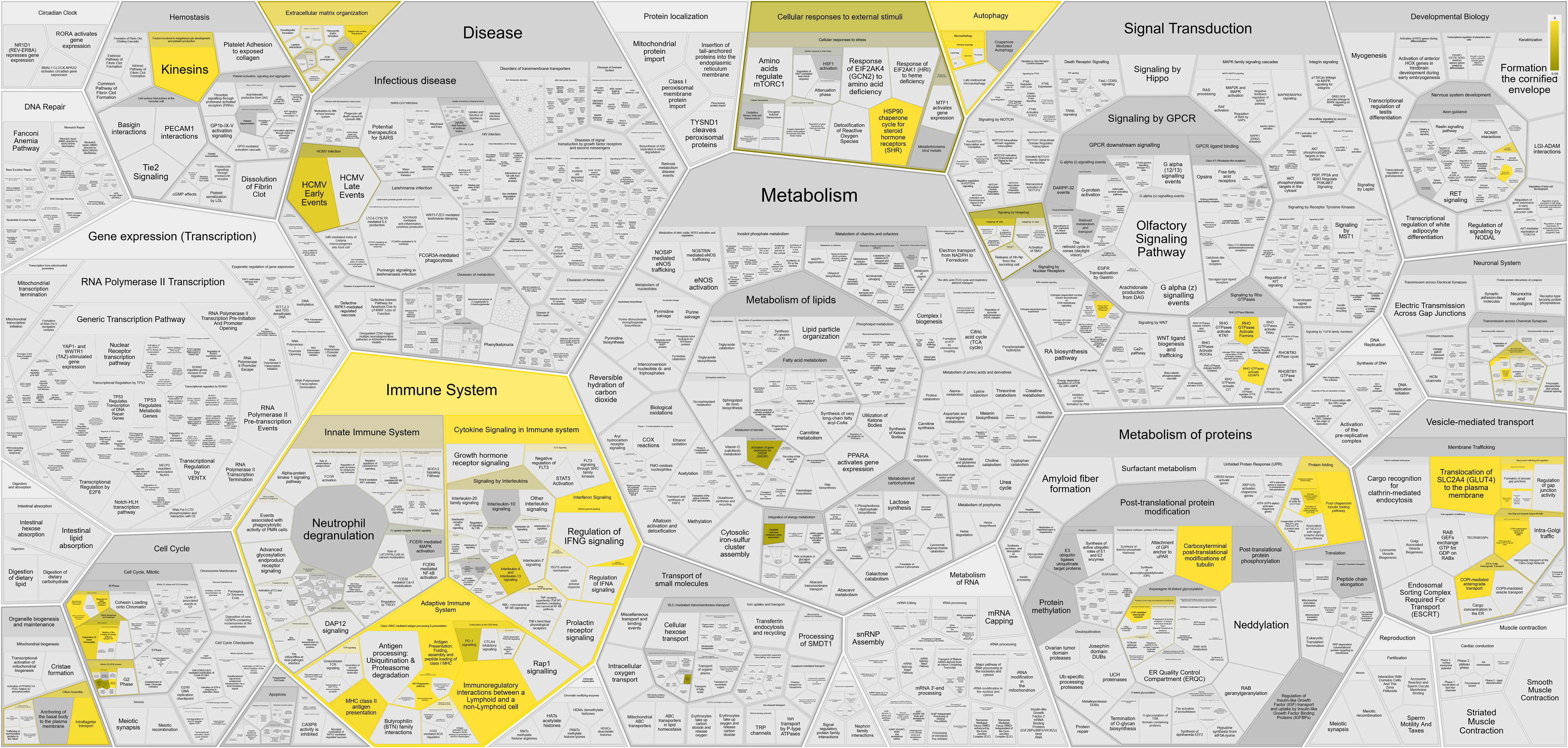

Supplement: Supplementary file 1 [file cells-10-00711-s001.zip › Supplementary/Supplementary Figure S1.tif]

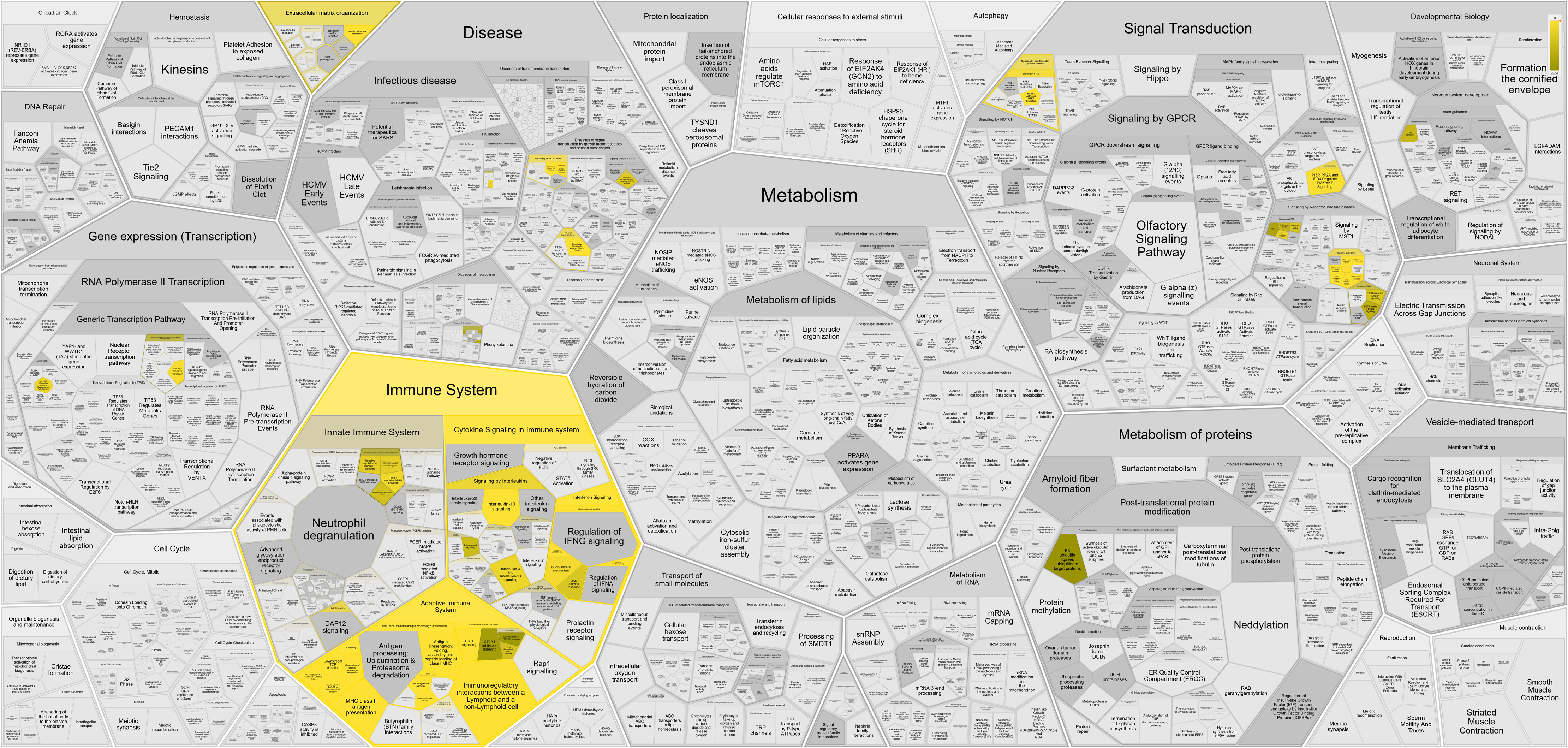

Supplement: Supplementary file 1 [file cells-10-00711-s001.zip › Supplementary/Supplementary Figure S2.tif]
